# Electron-to Hole Transport Change Induced by Solvent Vapor Annealing of Naphthalene Diimide Doped with Poly(3-Hexylthiophene)

**DOI:** 10.3389/fchem.2021.703710

**Published:** 2021-08-05

**Authors:** Krzysztof Janus, Kinga Danielewicz, Dorota Chlebosz, Waldemar Goldeman, Adam Kiersnowski

**Affiliations:** ^1^ Department of Physical and Quantum Chemistry, Faculty of Chemistry, Wroclaw University of Science and Technology, Wroclaw, Poland; ^2^ The Leibniz Institute of Polymer Research, Dresden, Germany

**Keywords:** naphthalene diimide, doping, charge carrier mobility, naphthalene diimides, poly (3-hexylthiophene)

## Abstract

Herein we report on fabrication and properties of organic field-effect transistors (OFETs) based on the spray-coated films of N,N′-dioctyl naphthalene diimide (NDIC8) doped with 2.4 wt% of poly (3-hexylthiophene) (P3HT). OFETs with the untreated NDIC8:P3HT films revealed electron conductivity [μ_e_* = 5 × 10^–4^ cm^2^×(Vs)^−1^]. After the annealing in chloroform vapor the NDIC8:P3HT films revealed the hole transport only [μ_h_* = 0.9 × 10^–4^ cm^2^×(Vs)^−1^]. Due to the chemical nature and energy levels, the hole transport was not expected for NDIC8-based system. Polarized optical- and scanning electron microscopies indicated that the solvent vapor annealing of the NDIC8:P3HT films caused a transition of their fine-grained morphology to the network of branched, dendritic crystallites. Grazing incidence wide-angle X-ray scattering studies indicated that the above transition was accompanied by a change in the crystal structure of NDIC8. The isotropic crystal structure of NDIC8 in the untreated film was identical to the known crystal structure of the bulk NDIC8. After the solvent annealing the crystal structure of NDIC8 changed to a not-yet-reported polymorph, that, unlike in the untreated film, was partially oriented with respect to the OFET substrate.

## Introduction

Scalable wet deposition technologies, such as spray-coating, gravure, slot-die coating, meniscus-guided coating etc. are considered amongst the most important, technologically-relevant strategies of fabrication of organic, thin-film based electronic devices (Hwang, Bae, and Kim 2014; Koutsiaki et al., 2019; Chen et al., 2020; Chaturvedi et al., 2021; Michels et al., 2021). Formation of the solid films upon any wet deposition process can be considered a problem of a crystallization of solution components at the solid surface. In order, therefore, to fully understand and further develop wet deposition technologies, in depth studies on solution crystallization—at the solid-liquid interface are necessary (Michels et al., 2021).

Electronic devices based on binary blends may reveal unique properties due to doping or formation of phase separated morphologies with desired architecture (Sepe et al., 2014; Afraj et al., 2021). Blending of p-type conjugated polymers with n-type small molecules may result in formation of bulk heterojunctions (BHJ). Control over formation of BHJ can be used to tailor properties of light-emitting diodes and photovoltaics or achieving balanced transport of electrons or holes in ambipolar organic field-effect transistors (OFETs) (Wang and Yan 2010; Puniredd et al., 2013). Wet fabrication of electronic devices based on binary systems deserves even more attention for its particular complexity. When two components crystallize from a common solution, the differences in solubility induce differences in precipitation sequence. The sequential precipitation may lead to heterogeneous nucleation or even, relatively rarer epitaxy (Bu et al., 2012; Chlebosz et al., 2017).

Either heterogeneous nucleation or epitaxial growth may cause formation of metastable film morphologies that can be kinetically equilibrated by thermal or solvent vapor annealing (Chlebosz et al., 2020; Mori et al., 2020). Both kinds of annealing cause profound changes in film morphology (Schulz and Ludwigs 2017; Chlebosz et al., 2020; Fidyk et al., 2020). Solvent vapor annealing is process substantially longer than the film deposition. Hence, during the solvent vapor annealing the metastable, “kinetically frozen” crystal systems can recrystallize to form more stable morphologies. In addition, the solvent vapor annealing permits a better control over the nucleation and growth in the polycrystalline films (Schulz and Ludwigs 2017). Therefore this strategy was assumed here to stabilize thin films based on *N,N′*-dioctyl naphthalene diimide (NDIC8) doped with 2.4 wt% of poly (3-hexylthiophene) (P3HT). Core-unsubstituted naphthalene diimides (NDIs) were broadly reported as effective n-type semiconductors. Heterocyclic or aromatic extension of the naphthalene cores may, however, change their charge transfer characteristics from n-type to p-type by shifting NDIs’ energy levels (Suraru et al., 2011; Al Kobaisi et al., 2016). The motivation to study the blend of the core-unsubstituted NDIC8 doped with P3HT was, however, to further explore a potential of alkylated NDIs as n-type semiconductors in hybrid systems, as indicated in our previous works (Janasz et al., 2018). Previously we have demonstrated that adding small quantities of NDIs in P3HT can enhance hole transport in the P3HT:NDI blends (Chlebosz et al., 2020). The goal in this study was to test the hypothesis if doping of NDIC8 with small quantities of p-type polymer (P3HT) can enhance electron transport in the way analogical as observed for p-type P3HT-based systems (Chlebosz et al., 2020).

## Materials and Methods

### Materials

N,N′-di (n-octyl)naphthalene-1,4,5,8-tetracarboxylic diimide (NDIC8) was synthetized according to protocols available in literature (Ke et al., 2015). Poly (3-hexylthiophene) (P3HT) with molar mass 65.2 kg/mol and regioregularity 95.7% was purchased from Ossila (Sheffield, United Kingdom). Chloroform (HPLC grade Chromasolv® 34,854) was obtained from Sigma-Aldrich. Solutions of NDIC8:P3HT were prepared by dissolving NDIC8 in previously prepared chloroform solution of P3HT (1 mg/ml) to obtain weight ratio NDIC8:P3HT equal to 40:1.

### Samples Preparation

NDIC8:P3HT solutions were spray-coated on silicon and glass substrates at 25°C. Dry nitrogen was used as a carrier gas. In the study, a computer-controlled, custom-built spray-coater equipped with precise nozzle (IWATA) was used. The nozzle-to-substrate surface distance was set to 70 mm. Approximately 0.125 ml of NDIC8:P3HT solution was used to prepare a set of two samples in a single run. One sample was studied directly after spraying without any further treatment, the second was solvent annealed. The film thickness was determined by atomic force microscopy. Directly after the spray-coating the films were relatively continuous and had thickness of approx. 700 nm. After the solvent vapor annealing the film roughness significantly increased due to formation of the coarse needle-like crystals. That coarsening made precise determination of the film thickness not possible. Analysis of the height profiles suggested that the thickness of the solvent vapor annealed films can reach as high as approx. 2,200 nm measured from the substrate surface to the fibre top. Height profiles are included in the supporting information (SI, see Supplementary Figure S1). The solvent annealing was performed by placing the spray-coated samples for 24 h in chloroform vapor under ambient pressure (∼10^5^ Pa) at 25°C. Transistors were prepared using silicon substrates (15 × 20 mm) with 300 nm silicon dioxide dielectric layer (Ossila). Electrically characterized samples were further investigated using GIWAXS and polarized optical microscopy. For direct observations of morphology transitions occurring during the solvent vapor annealing, the films were spray-coated on glass/quartz substrates (15 × 20 mm, Ossila) using the same parameters as for fabrication of transistors.

### Charge Transport Characterization

In order to measure electrical characteristics of NDIC8:P3HT blend, they were used as active films in OFETs. The OFETs were fabricated by evaporation of 80 nm-thick gold source and drain electrodes on previously prepared NDIC8:P3HT films deposited on silicon/SiO_2_ substrates. In this way bottom gate, top contact transistors were fabricated. The channel width was constant and equal to 1,000 μm whereas the channel length was varied within the range of 10–80 μm. Measurements were performed under oxygen and water free atmosphere in a glove box. Prior to characterization the samples were stored in the glove box for at least 24 h. Output and transfer characteristics were taken with Keithley 4200 Semiconductor Characterization System. The voltages were ranged from 10 to −80 V for hole conductivity measurements and −10 to 80 V for electron conductivity. Transfer characteristics were recorded for drain-source voltages equal to −80 and 80 V, respectively; their slope was used to determine charge carriers mobility. For all the samples both electron and hole characteristics were examined.

### X-Ray Scattering Measurements

Grazing-incidence wide angle X-ray scattering (GIWAXS) experiments were performed at the P03/MINAXS beamline at Petra III synchrotron facility in Hamburg, Germany. In the GIWAXS experiments the microfocused X-ray beam with energy of 12.85 keV (λ = 0.965 Å) was used. For each sample, the incident angle (α_i_) was set to 0.1°. The scattered intensity was recorded on a Dectris Pilatus 300 k detector within the 0.1 s exposure time. Examination of samples after the measurements revealed that such exposure did not cause any beam damage. Data processing was performed using Datasqueeze 3.0 and Origin 2016 computer programs.

### Polarized Optical Microscopy

The polarized optical micrographs were taken using Olympus BX-60 microscope equipped with crossed polarizers and Panasonic DMC-G2 camera. Movies showing the phase transition were composed from time-lapse sequence of still images taken with 60–120 s intervals.

### Scanning Electron Microscopy

The samples were prepared as described in “samples preparation” paragraph. Samples were not metallized before imaging in the SEM in order to keep the natural surface and all kinds of native contrasts accessible. Images, providing topographic and material contrasts, were acquired using the LEO Gemini 1,530 field-emission scanning electron microscope at landing voltages in the range from 100 to 200 V by using the in-column ring detector (Inlens-SE) at working distance of around 2 mm. In order to minimize the charging of the samples, the scan was performed as line averaging (2048 × 1,536 over 22.7 s). Similar to the herein adopted imaging methodology was successfully used to analyze photovoltaic polymer blends (Masters et al., 2017).

## Results and Discussion

**FIGURE 1:**
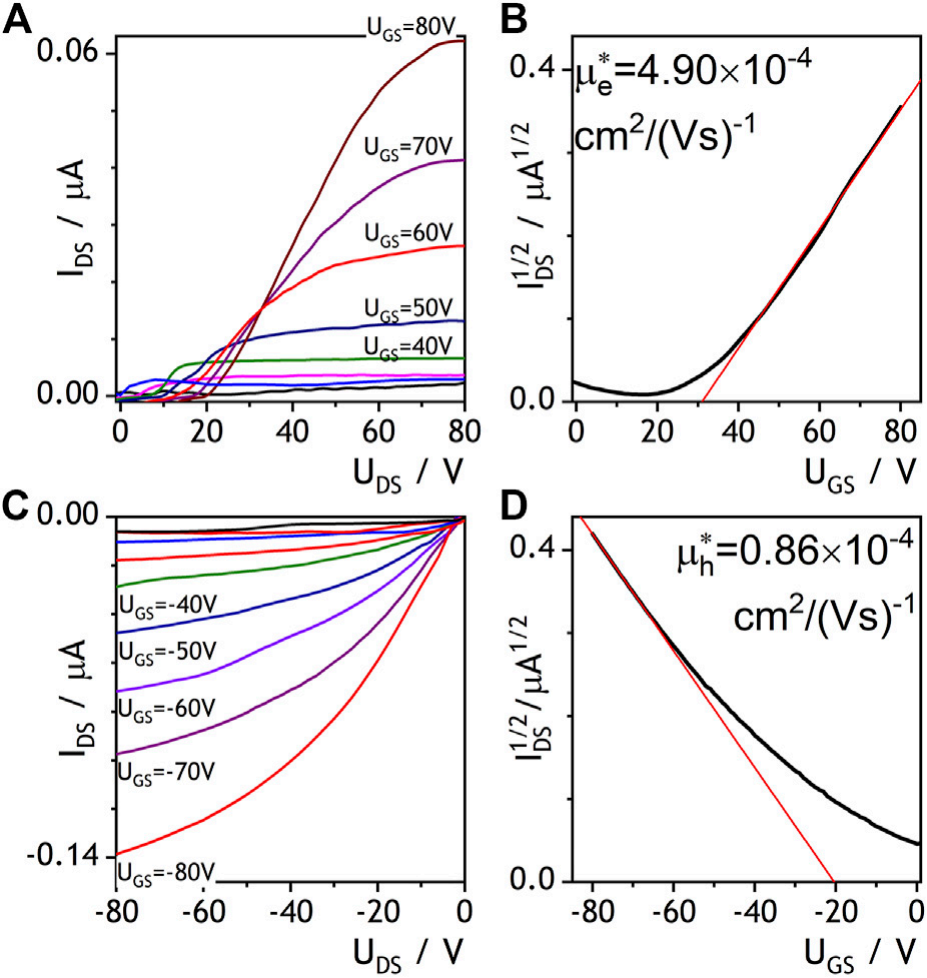
OFET characteristics measured for NDIC8:P3HT blend before **(A, B)** and after **(C, D)** solvent vapor annealing. Output curves are shown in panels **(A)** and **(B)**, transfer curves are displayed in panels **(C)** and **(D)**.

**FIGURE 2:**
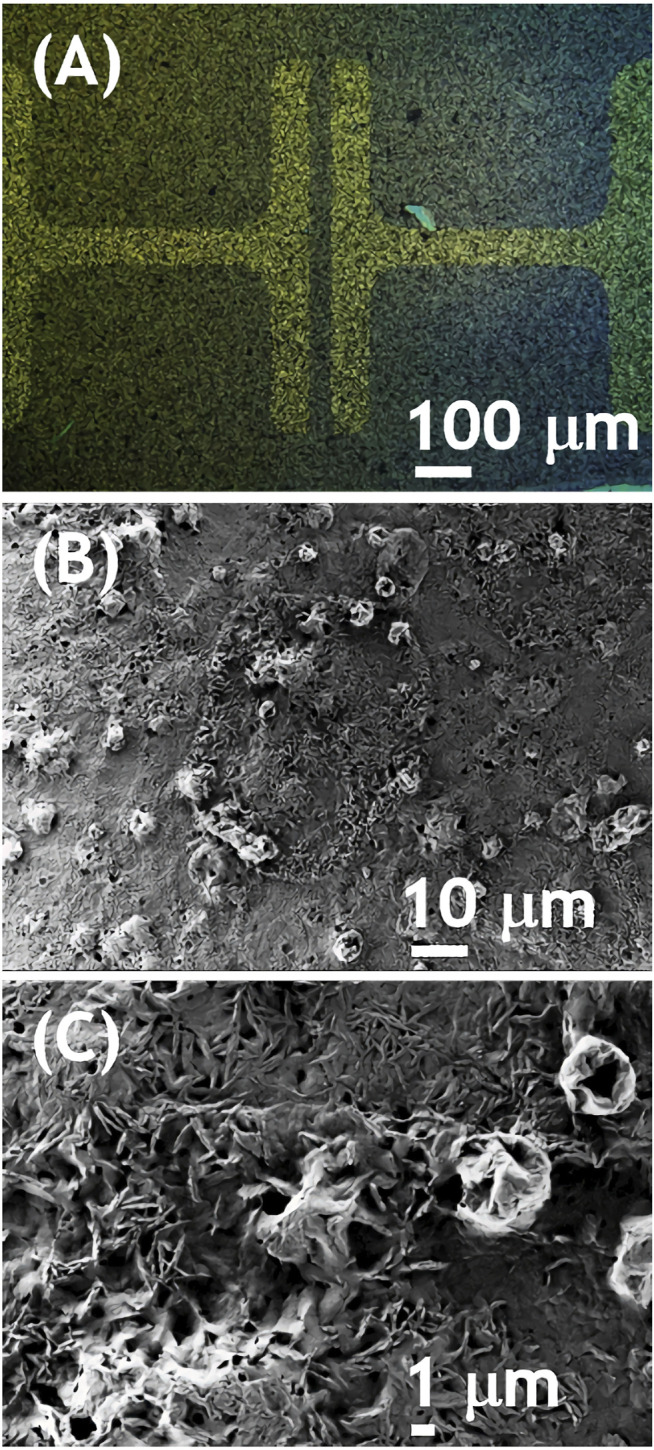
Optical **(A)** and scanning electron **(B, C)** micrographs of NDIC8:P3HT spray-coated on an OFET substrate.

As outlined in the introduction, our goal was to verify, whether doping an electron conducting NDIC8 with hole conducting P3HT has an impact on the electron conductivity. The reason to focus the study on the NDI derivative was mainly a proven air stability enabling processing under ambient atmosphere (Jung et al., 2009). Hence, NDIC8, a representative example of NDIs, was tested in thin-film field effect transistors (OFETs). In accordance with our expectations based on literature reports (Ichikawa et al., 2013; Ma et al., 2016) NDIC8 revealed a moderate electron conductivity (OFET data not shown in this work). Our measurements revealed that electron mobility (μ_e_) in pure NDIC8 equaled 53.4 × 10^–4^ cm^2^×(Vs)^−1^ that was lower than the maximum mobilities reported in literature (Ma et al., 2016). The lower, in comparison to literature data, μ_e_ recorded in our study most likely stemmed from non-optimal selection of transistor geometry and a mismatch between LUMO level of NDIC8 and work function of gold electrodes. The solvent vapor annealing of NDIC8 caused a reduction of the μ_e_ down to 0.25 × 10^–4^ cm^2^×(Vs)^−1^, which we have attributed to dewetting of the film, coarsening of the crystal morphology (cf. Supplementary Material) and hence reduction in the effective cross-section of the OFET channel observed in the optical micrographs (see Supplementary Figure S2 in the supporting information, SI). The NDIC8:P3HT blend directly after the spray coating revealed electron conductivity as well (Figures 1A,B). The output characteristics of NDIC8:P3HT showed typical for OFETs profile slightly disturbed at low drain-source voltages. We have attributed the non-ideal profiles to high contact resistance related to the aforementioned mismatch of gold electrodes and LUMO level of NDIC8 and formation of Schottky barrier (Horowitz 2010). In spite of non-ideal output, transfer characteristics measured at high U_DS_ (80 V) was typical, and linear at high gate-source voltages. This is not uncommon, but determining the charge carriers mobility from the slope of this linear section may be strongly over or underestimated (Un, Wang, and Pei 2019). Therefore, it was only possible to determine estimated electron mobility from the slope of that linear section. In order to indicate that estimation, we have used the symbol of μ_e_*. The average μ_e_* equaled 5 × 10^–4^ cm^2^×(Vs)^−1^ at no hole conductivity. Micrographs, both taken with polarized (POM) and scanning electron microscopes (SEM), revealed a granular morphology of the spray-coated NDIC8:P3HT films (Figure 2). A closer inspection of the morphology (Figure 2C) indicated the existence of small, elongated NDIC8 crystals present throughout the film (i.e., both in granules visible in POM and in between the granules). Such a morphology is favorable for percolation paths and therefore enabled electron transport through the NDIC8:P3HT films. Our X-ray scattering results (discussed later in the work) suggested that the NDIC8:P3HT films before the solvent vapor annealing can be considered a dense network of pure NDIC8 nanocrystals.

Solvent vapor annealing of NDIC8:P3HT-based OFETs caused electron conductivity to vanish. We have initially attributed electron conductivity loss to dewetting and formation of larger, but separated crystal domains and, consequently, breaking percolation paths in the film. However, testing OFETs with negative gate voltages indicated that the NDIC8:P3HT blend after the solvent vapor annealing revealed a surprising hole conductivity (Figures 1C,D). The hole mobility (μ_h_*) estimated from OFET characteristics (which again were non-ideal due to high drain current at zero gate voltage, what may be attributed to e.g., unintentional doping with atmospheric oxygen) was found 1 × 10^–4^ cm^2^×(Vs)^−1^, i.e., was approx. five-fold lower than the μ_e_* determined for the blend before solvent vapor annealing. Note, however, that the origin of a hole transport in the NDIC8:P3HT, even if unimpressive, is nontrivial, since the amount of P3HT the only hole-transport-capable component—was as low as 2.4 wt%.

**FIGURE 3:**
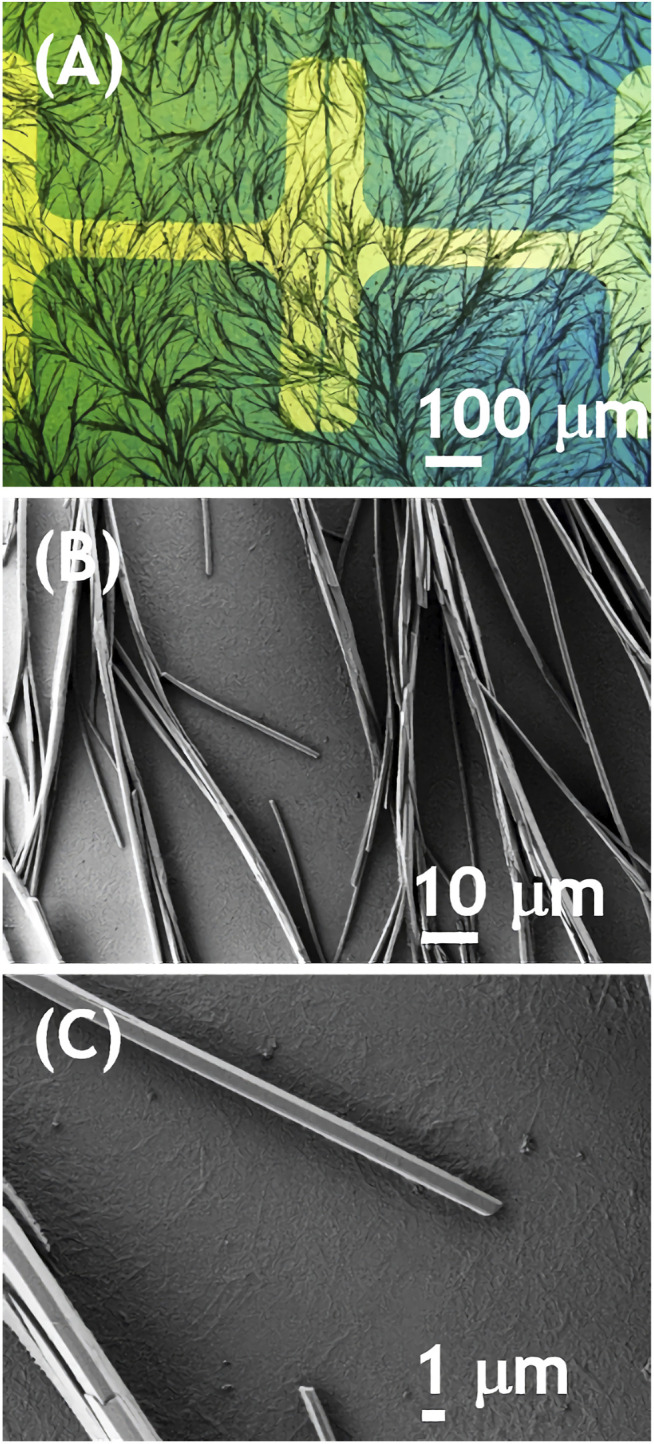
Optical **(A)** and scanning electron **(B, C)** micrographs of P3HT:NDIC8 blend spray-coated and solvent annealed on an OFET substrate.

In order to explain this observation we have analyzed morphology and crystal structure of the films. The process of recrystallization upon solvent vapor annealing was directly observed with polarized light microscope. The solution was spray coated on transparent substrate and placed in set of chloroform-vapor-filled Petri dishes in optical path of microscope. A movie showing the recrystallization is available in the SI. The transition from granular to a remarkable dendritic morphology took approximately 40 min. Static micrographs of NDIC8:P3HT films (Figure 3) showed a dendritic morphology formed by needle-like crystals with approx. 1 μm in width and length reaching tens of μm (Figures 3B,C). Note that such crystals were not formed by pure NDIC8 that after the solvent vapor annealing formed clear monoclinic or rhomboid crystals (see Supplementary Figure S1 in SI). Moreover, a close inspection of Figure 3C revealed that a layer exhibiting fine fibrillar morphology underpinned the layer of large, needle-like crystals.

**FIGURE 4:**
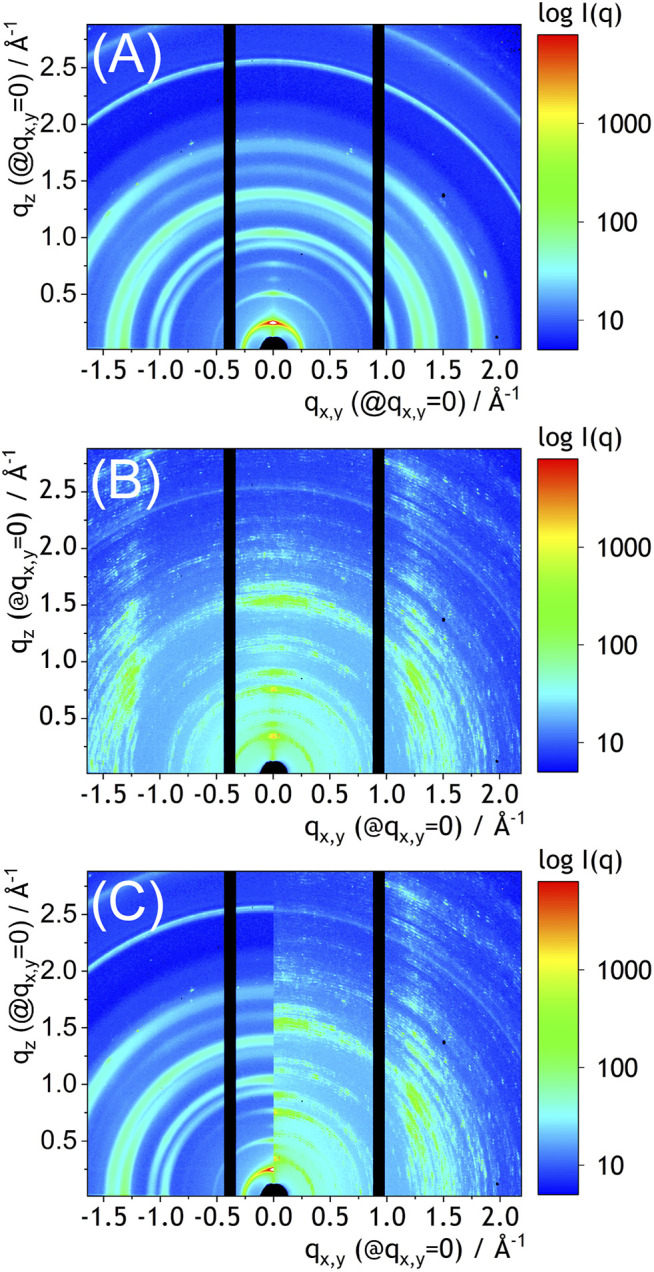
GIWAXS patterns (12.85 keV) of the sprayed coated NDIC8:P3HT film **(A)**, the sprayed-coated and solvent annealed NDIC8:P3HT film, and their comparison in the common coordinate system **(C)**. Note that the patterns represent unwarped, non Fraser-corrected detector data. Hence, the q_z_ and q_x,y_ are precise only at q_x,y_ = 0 and q_z_ = 0. At nonzero q_x,y_ for q_z_ and q_z_ for q_x,y_ the q-scale is approximate.

In order to further examine the role of solvent vapor annealing on properties of NDIC8:P3HT films they were analyzed by GIWAXS. Our GIWAXS studies (Figure 4) confirmed, that the crystal structure of NDIC8 in the NDIC8:P3HT blend before the solvent vapor annealing was identical with that of the pure NDIC8 (Figure 4A) (Jiao et al., 2019). The GIWAXS pattern of NDIC8:P3HT after the solvent vapor annealing (Figure 4B) was, however, notably different from the pattern recorder before the solvent annealing (Figure 4C). The characteristic, speckled pattern resulted from the formation of large, non-randomly oriented crystal domains, which was evidenced by microscopy (Figure 3). More importantly, however, the positions of GIWAXS peaks observed for the solvent vapor annealed NDIC8:P3HT were significantly different from those observed for the blend directly after the spray-coating. A clear shift of the main peaks becomes more evident when the GIWAXS patterns are directly compared in a common q_x,y_/q_z_ coordinate system (Figure 4C). The azimuthal intensity distribution visible in Figure 4B indicates that the crystals formed as a result of solvent vapor annealing were partially oriented towards the substrate surface. The intensity distribution over the q scale indicates that solvent-vapor annealing resulted in formation of a new crystal phase. The positions of peaks recorded in this study cannot be attributed to any known structure of neither NDIC8 nor P3HT. The small amount (2.4 wt%) and nature of P3HT crystals disables considering the polymer as a direct cause of distinct, intense peaks visible in the pattern shown in Figure 4B. Unfortunately without a detailed crystallographic studies, we can only hypothesize that the diffraction peaks observed in Figure 4B originate from a not-yet-identified polymorph of NDIC8 or, which is rather less likely, formation of the NDIC8:P3HT cocrystals.

## Conclusion

Our study demonstrates that unlike in the case of p-type polymers, charge carrier transport in n-type materials, such as naphthalene diimides (NDIC8 here), cannot be enhanced by doping with small amounts of p-type polymers (P3HT here). Directly after the film fabrication the pure NDIC8 and the reported here NDIC8:P3HT system revealed, expectedly, electron conductivity. Adding the P3HT to NDIC8 caused, however, a reduction of electron mobility by approx. 1 order of magnitude. Adding as small as 2.4 wt% of P3HT in NDIC8 caused a massive change in recrystallization behavior of NDIC8 observed during the solvent vapor annealing. Whereas the NDIC8 recrystallized upon chloroform vapor annealing grown to form regular monoclinic-like crystals, in the NDIC8:P3HT blend formation of dendritic morphologies consisting of needle-like NDIC8 crystals was observed. Analysis of the static electron- and time-lapse optical micrographs suggests that solvent vapor annealing caused a complete reorganization of the film morphology by dissolving the existing and self-assembly of an entirely new crystal system in the film. Our results suggest that formation of the new crystal system was based on nucleation and growth. Such a recrystallization mechanism is different than typically reported Ostwald-ripening-based growth of crystal domains in the film (Chlebosz et al., 2017; Fidyk et al., 2020). The crystal structure of needles forming the NDIC8:P3HT dendritic morphology was not previously reported in the literature and requires further studies. Unlike the pure solvent-vapor recrystallized NDIC8, the P3HT-doped, solvent-vapor recrystallized NDIC8 exhibited an apparent hole conductivity that was never reported in literature for the class of core-unsubstituted NDI molecules. Our GIWAXS results suggest that the reassembly of NDI molecules in the presence of P3HT results in formation of the new, yet-unknown crystal phase. The role of P3HT on formation of that crystal phase is, however, unclear and requires further studies. Probably, it would be necessary to trace the phase transition with a time-resolved method, such as time-resolved X-ray scattering. It is also unclear if packing of NDIC8 molecules in the new crystal phase can be the reason behind changing the charge transport mode from n-type to p-type. Similar example of “switching” from electron to hole transport was reported for the thermally annealed blend of poly {2,5-bis(3-tetradecylthiophene-2-yl)thieno [3,2-b]thiophene} (PBTTT) with one of perylene diimide (PDI) derivatives mixed at the 1:1 ratio (Fidyk et al., 2020). The change in the charge transport mode in the PBTTT:PDI blend was attributed to a morphological change caused by thermally-induced crystal growth and a consequent phase separation during the thermal annealing. In this work, it seems that the change in charge transport mode was based on a different mechanism, mainly because of the completely different blend compositions in the cited and our studies. Regardless of the mechanism, it seems that switching the charge transport mode upon annealing is characteristic of some polymer-small molecule blends and should be explained in more extensive studies.

## Data Availability

The original contributions presented in the study are included in the article/Supplementary Material, further inquiries can be directed to the corresponding authors.
