# Supplementary material for: Electron-to Hole Transport Change Induced by Solvent Vapor Annealing of Naphthalene Diimide Doped with Poly(3-Hexylthiophene)
Source: Front Chem. 2021 Aug 5;9:703710. doi: 10.3389/fchem.2021.703710 (PMC8375403; doi:10.3389/fchem.2021.703710)
Supplement: Supplementary file 2 [file datasheet1.pdf]

Electron- to hole transport change induced by solvent vapor annealing of naphthalene diimide doped with poly(3-hexylthiophene)

Krzysztof Janus[1,2*†], Kinga Danielewicz[1], Dorota Chlebosz[1,2], Waldemar Goldeman[1], Adam Kiersnowski[1,2*†]

[1] Wroclaw University of Science and Technology, Wybrzeze Wyspianskiego 27, 50-370 Wroclaw, Poland

[2] The Leibniz Institute of Polymer Research, Hohe Str. 6, 01069, Dresden, Germany

Supplementary Material

1 Supplementary Figures

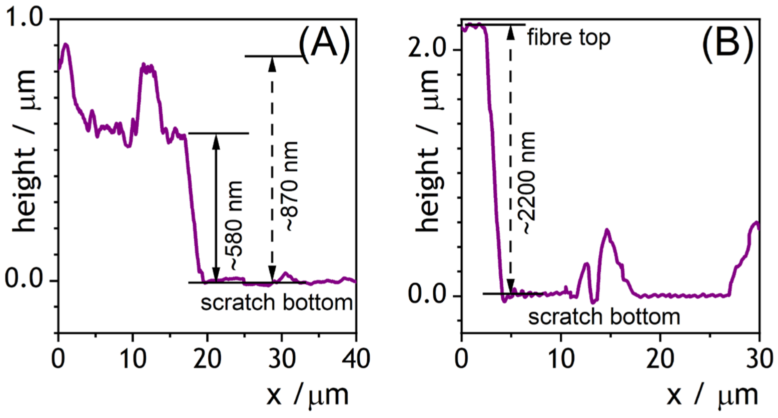

Supplementary Figure SF1. AFM height profiles of films scratched with needle tip. (A): full substrate-top height profile of the film prior to the solvent vapor annealing, (B): full substrate-top height profile of the film after the solvent vapor annealing.

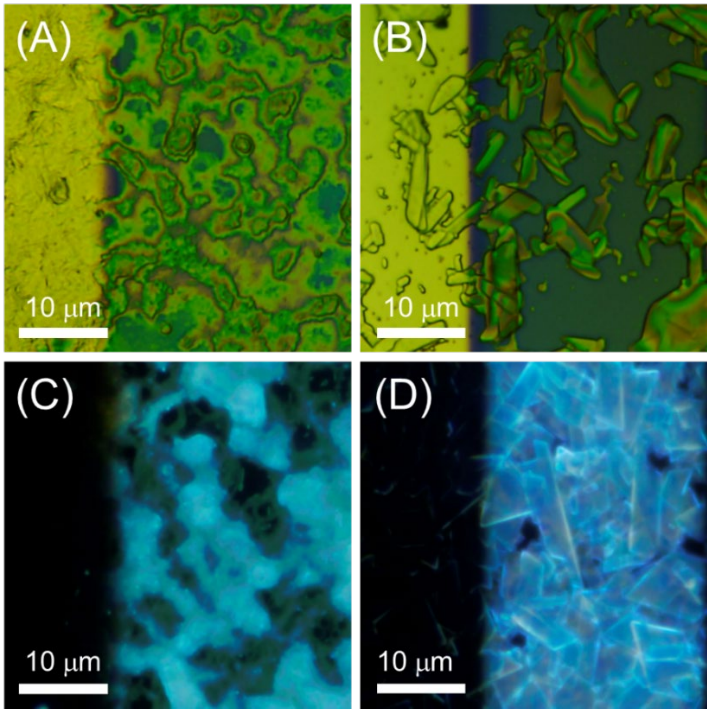

Supplementary Figure SF2. Microscopic polarized (A), (B) and fluorescence (C), (D) images of NDIC8 transistor channels before (A), (C) and after solvent annealing (B), (D).

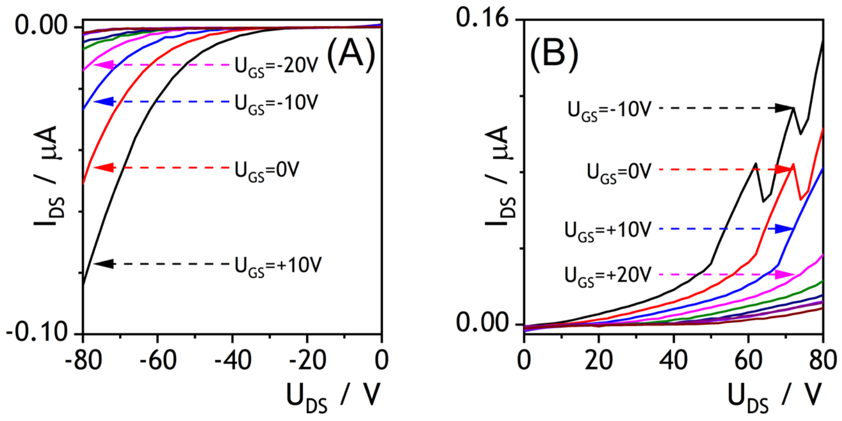

Supplementary Figure SF3. Output OFET characteristics measured for NDIC8:P3HT blend before (A), and after (B) solvent vapor annealing showing the absence of, respectively, hole and electron conductivity.
